# Next-Generation PVA–Dye Complex Film with Advanced Properties for Optical Applications

**DOI:** 10.3390/polym18070876

**Published:** 2026-04-02

**Authors:** Rong Ma, Yuncheng Yu

**Affiliations:** Artie McFerrin Department of Chemical Engineering, Texas A&M University, College Station, TX 77843, USA; yyc@tamu.edu

**Keywords:** polyvinyl alcohol, film, thermal stability, water resistance, optical applications

## Abstract

With the development of the information society, display technologies are evolving toward greater flexibility and advanced performance. Dye-based polyvinyl alcohol (PVA) complex films have gained widespread attention for their excellent resistance to high temperature and humidity. This review systematically summarizes the research progress of dye-based polarizers using PVA as the substrate, focusing on their preparation principles, film properties, and impacts on optical performance. Strategies to enhance optical properties and durability are discussed, including dye molecular optimization, formulation design, control of the dyeing process, and PVA substrate film modification. Notably, improving interfacial interactions between dyes and PVA enhances molecular orientation and stability, while PVA modification improves mechanical properties, water resistance, thermal stability, and flame retardancy. By demonstrating these enhanced comprehensive properties, this review highlights the potential of PVA–based films to serve as high-performance platforms for the development of next-generation multifunctional optical and display materials. Finally, the challenges and development directions of dye-based PVA complex films for optical applications in harsh environments are prospected. This review provides a theoretical basis and technical pathway for the design and development of next-generation high-performance composite polarizers.

## 1. Introduction

With the development of the information society, display technology has permeated various fields of daily life. Display technologies are evolving toward lighter, thinner, and more flexible formats with high optical performance and durability. Currently, the mainstream display technologies are Thin-Film Transistor Liquid Crystal Displays (TFT-LCDs) and Organic Light-Emitting Diode (OLED) displays. In these display systems, polarizers are an essential component. In LCD panels, two polarizers are required for image formation. The bottom polarizer converts natural backlight into polarized light, while the top polarizer controls the transmission of light to create bright and dark states. In OLED panels, only one polarizer is needed to eliminate external ambient reflections and provide a high-contrast display [1]. As a key component of display technology, understanding the influence of each component of the polarizer and the relevant factors in the processing is crucial for developing next-generation large and flexible display panels.

**Figure 1:**
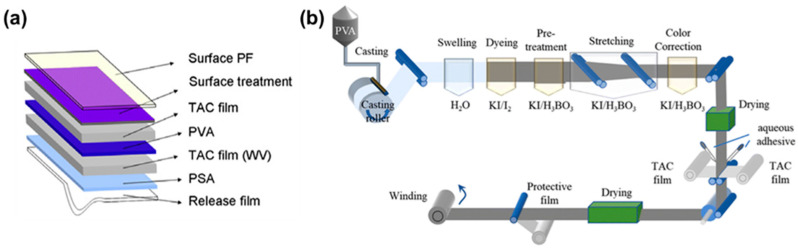
(**a**) The layered structure of a typical polarizer (reprinted with permission from Ref. [3], Elsevier, 2011); (**b**) the main preparation process of a polarizer [4] (reprinted with permission from Ref. [4], Royal Society of Chemistry, 2025).

The most common polarizer is the H-type polarizer, which is a common and inexpensive type of linear polarizer made from stretched polyvinyl alcohol (PVA) with dichroic materials. A polarizer typically consists of a polarizing film, a support film, a pressure-sensitive adhesive (PSA), and other components. As shown in Figure 1a, the protective film (PF) is primarily used to prevent damage to the polarizers during cutting and transportation processes. Triacetyl cellulose (TAC) film is mainly used to block moisture to maintain structural and dimensional stability of the PVA polarizing film under different temperature and humidity conditions. They are laminated on both sides of the PVA polarizing film. The PVA polarizing film is typically fabricated by applying polyvinyl alcohol (PVA) as the substrate and iodine or dyes as the dichroic materials [2]. It determines the key optical performance of the polarizer, such as polarizing efficiency (PE), transmittance, and hue. The PSA layer is used for bonding the polarizer to the panel. The release film is located at the bottom of the polarizer to protect the PSA layer [3]. As shown in Figure 1b, the main manufacturing process of PVA–based polarizer films involves the stretching and lamination of the PVA polarizing film. It includes swelling, dyeing, crosslinking, and stretching. This stage causes the PVA molecular chains to change from disordered to highly ordered along the machine direction. Meanwhile, the polyiodide ions or dye molecules become ordered to generate polarized light macroscopically. The next stage mainly involves coating and laminating release films to produce the final polarizer [3,4,5,6].

In the fabrication of polarizers, the film formation mechanism of PVA film and the influence of the interaction between various components during the preparation processing have aroused great interest. There has been extensive research and reviews on iodine-based polarizers. This review primarily summarizes the research progress related to dye-based polarizers. This article mainly introduces research work related to PVA film manufacturing and summarizes the work in improving PVA–dye complex optical properties. Finally, this review presents prospects and viewpoints on the improvement in the preparation and processing of PVA–dye complex film.

## 2. PVA–Dye Complex Film Preparation Process and Mechanism

**Figure 2:**
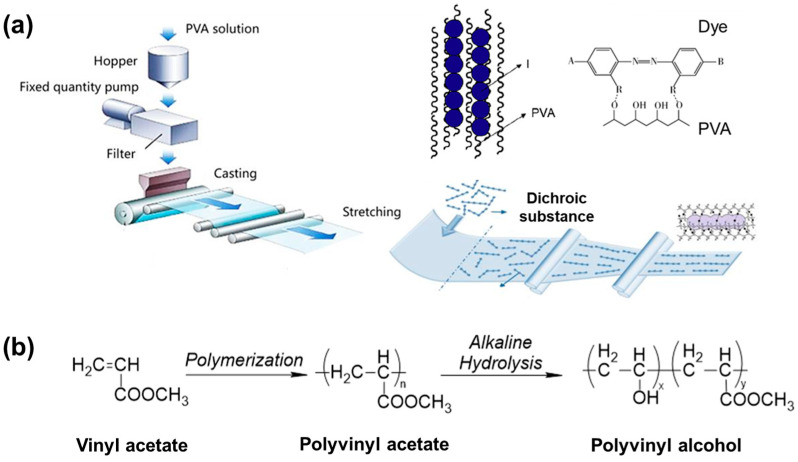
(**a**) The preparation of PVA optical film and the binding diagram of polyiodide molecules or dyes in PVA–based film (reprinted with permission from Ref. [3], Elsevier, 2011.) (**b**) Synthesis process of PVAc and PVA.

Polarizers are made from oriented PVA containing polyiodide or dichroic dye as dichroic materials. By stretching PVA film, the dichroic materials, such as azo dyes, become aligned with the substrate during this process, resulting in anisotropic absorption of light (Figure 2a). This leads to a typical characteristic of polarizers: there is no light transmission through two orthogonal polarizers, while two parallel polarizers exhibit high transmittance [7,8].

### 2.1. Characteristic Properties of PVA Film

The chemical and physical properties of the PVA film play a crucial role in the production of the polarizing film. The chemical structure, including the molecular weight and distribution of PVA, the degree of hydrolysis of PVA chains, sequence length, and the content and distribution of 1,2-diol units, will significantly affect the binding behavior of PVA chains with dichroic materials and their orientation performance during stretching [9,10]. These factors directly influence the optical performance of the PVA–based polarizing film.

The industrial synthesis of PVA polymers involves free radical polymerization as described in Figure 2b. In brief, vinyl acetate is polymerized to obtain polyvinyl acetate (PVAc) and then hydrolyzed to obtain PVA [11,12]. However, it is difficult to precisely control the chain structure during the free radical polymerization process. It can lead to various structures and defects in the PVAc chains. These defects affect the final structure and performance of the final PVA optical film and polarizing film. Current research results indicate that PVA is mainly composed of head-to-tail connected 1,3-diol units, wherein the hydroxyl side groups are positioned on alternating carbon atoms of the main chain. The presence of these 1,2-diol structures would hinder the formation of a PVA–iodine complex and affect the crystallization of PVA [13,14,15]. When there are too many 1,2-diol units in the PVA chain, it prevents the formation of the characteristic blue-violet PVA–iodine complex. Simultaneously, the syndiotactic structure of PVA also more readily forms complexes with iodide ions [13,14]. Furthermore, the tacticity of PVA significantly affects its solubility in different solvents, thermal stability, mechanical properties, and processability [16]. Some publications have shown that highly isotactic PVA has better ductility than highly syndiotactic PVA because syndiotactic sequences more easily form hydrogen bonds between PVA molecular chains, which hinder the stretching of PVA chains [17]. PVA is obtained by hydrolysis of PVAc under alkaline conditions. The degree of hydrolysis significantly affects the physicochemical properties of PVA. With an increasing degree of hydrolysis, PVA provides higher water solubility, crystallinity, and mechanical properties. Conversely, due to the amorphous nature of PVAc, PVA with a low degree of hydrolysis shows excellent moisture resistance and toughness but reduced thermal stability. Work from Wang et al. indicates that PVA with different degrees of hydrolysis forms different complexes with iodide ions. For example, the PVA–iodine complex formed by PVA with a low degree of alcoholysis (88%) is similar to that of pure PVAc, which affects the final polarizer performance [18].

In the industrial manufacturing process, PVA films are usually produced by casting from a PVA aqueous solution. Firstly, this process involves dissolving PVA resin, glycerol, and surfactants in water at high temperature and high pressure to form a uniform and high-concentration solution. Subsequently, the solution is extruded onto casting rolls and dried to form films. Initial shaping is conducted on the first casting roll, followed by drying and annealing treatments to enhance the internal structure of the film and improve its dimensional stability. Current research findings indicate that kinetic factors such as the solvent evaporation rate and cooling rate usually play key roles in the industrial casting process [19]. Rapid solvent evaporation drives the separation of crystalline and amorphous phases in PVA, but when the solvent evaporation rate at the surface exceeds that of the bulk, a glassy layer forms at the air–solution interface to further hinder solvent volatilization [20,21]. In addition, the initial solution concentration is also found to affect the drying behavior. Studies have shown that a lower initial concentration facilitates the formation of a more uniform structure, while a higher initial concentration results in internal structural inhomogeneity [22]. Meanwhile, in order to meet the manufacturing performance of PVA films under different conditions, plasticizers of different types and amounts are added to adjust their properties. For example, as the glycerol content increases, the crystallization temperature and melting point of the PVA film show a decreasing trend, and toughness increases while the modulus decreases [23].

### 2.2. Properties of PVA–Azo Dye Complex Polarizing Film

To investigate the performances of prepared PVA complexing films, researchers typically measure the dichroic ratio (R), single-piece transmittance (Ts), PE, and degree of polarization (DOP). In some works, they used DOP the same as with PE [24]. These parameters are calculated based on the following equations [25,26,27]:

$$R=A_{∥}/A_{⊥}$$

where $A_{∥}$ represents the absorbance of the dyed polymer when illuminated by polarized light vibrating parallel to the stretching direction while $A_{⊥}$ signifies the absorbance with polarized light perpendicular to stretching.

$$T_{s}=(T_{∥}+T_{⊥})/2$$

$$PE={\left[(T_{∥}-T_{⊥})/(T_{∥}+T_{⊥})\right]}^{1/2}$$

$$DOP=(T_{∥}-T_{⊥})/(T_{∥}+T_{⊥})$$

where $T_{∥}$ and $T_{⊥}$ are the transmittances of the film superimposed on each other, parallel and perpendicular to the direction of the elongation of the film, respectively. The wavelength of measurement ranges from 380 to 780 nm.

Most commercial PVA polarizing films use iodine as the dichroic material. However, under high temperature and humidity conditions, the iodine component tends to be released from the PVA host film. To overcome this problem, the use of azo-type dyes as dichroic materials to prepare PVA–based polarizing films has been investigated. These prepared PVA–dye polarizing films showed improved durability. In order to test the durability measurement under high temperature and high humidity, these polarizing films of the same size were subjected to a constant temperature and humidity chamber for a certain number of hours. For example (Table 1), they could maintain performance for 1000 h at 105 °C or 85 °C × 85% RH. But these dye-based polarizing films have inferior transmittance and PE compared to iodine-based polarizing films [28].

Dye-based polarizing films are produced by dyeing the PVA film with dichroic dyes, and they are typically stretched in a boric acid solution. The properties of dye components are important in determining the final performance of polarizing films. Typically, the required properties include high dichroism, high solubility in water, excellent heat and humidity resistance, and strong affinity to polymers [26]. The common dye molecules are azo, anthraquinone, and cyanine types. The characteristic anisotropic chemical structure of these dye molecules is that they have a long and planar structure to obtain high dichroic properties [29,30]. Dye molecules with this characteristic structure can absorb light strongly along their long molecular axis and weakly along the short axis. This selective absorption property allows dye molecules to convert natural light into polarized light. Although a single dye molecule has directional absorption characteristics, the dye molecules need to be aligned along a specific direction to exhibit macroscopic dichroism. The alignment of the dye molecules could be achieved through stretching, and the alignment order is related to the PE. A high PE requires highly consistent alignment of dye molecules [28]. The transmittance is related to dye concentration and molecular absorption characteristics, often requiring a balance between transmittance and polarization.

In order to improve water solubility, dye molecules often contain hydroxyl and amino groups that can also interact with PVA molecules through hydrogen bonds or van der Waals forces [26]. When dye molecules bind to PVA via hydrogen bonds, they move along the direction of the PVA chains during stretching. When the long axis of the dye molecule is parallel to the stretching direction, light parallel to the stretching direction is strongly absorbed. The strong interaction of hydrogen bonds ensures that the dye molecules align synchronously with the orientation of PVA during stretching, thereby producing macroscopic polarization. Furthermore, degradation behaviors of a dye-based PVA complex film under harsh conditions might be attributed to factors such as dye photodegradation and structural relaxation of the polymer chains. However, most existing studies focus on phenomenological observations, and detailed mechanistic investigations on degradation and structure–performance relationships remain limited.

## 3. Modification Methods for PVA–Dye Complex Film

As a key optical component of liquid crystal displays, dye-based polarizers have recently undergone significant technological development driven by application scenarios with stringent weatherability requirements such as automotive displays and outdoor digital screens. However, the comprehensive optical performance of PVA–dye complex films still show a wide performance gap compared with traditional iodine-based products, which limits their applications. One reason is the dichroic properties of dye molecules. On the other hand, the alignment order of dye molecules in the PVA matrix has a decisive effect on the final optical performance. The alignment order of dye molecules would be affected by the dyeing process. For example, improper dye combination may reduce overall polarization efficiency due to secondary absorption effects between molecules. Current modification strategies for dye-based complex film include optimizations of dye molecular structures and improvements in the dyeing process to improve their optical performances [31,32,33]. In addition, the modification of a PVA–based film might provide additional properties for the complex film, such as water resistance and flame retardancy.

### 3.1. Design of Dye Molecular Structure

**Figure 3:**
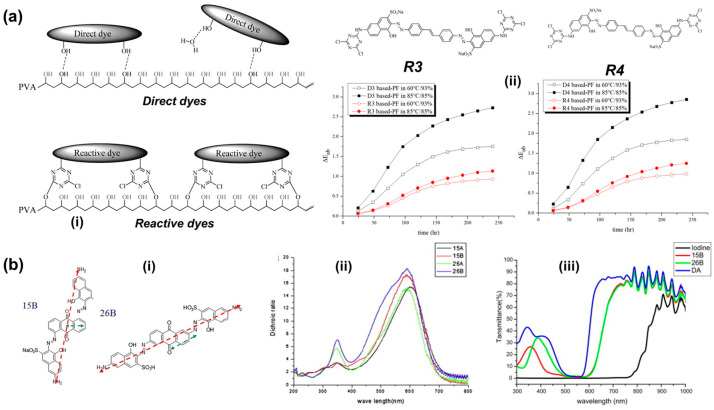
(**a**) Scheme of interactions between dyes and PVA for direct and reactive dye-based polarizing film (**i**), chemical structure of reactive dyes (R3, R4) and durability of the prepared polarizing films under high-temperature and humid environments for prepared films with direct dyes (D3, D4), R3 and R4 (**ii**) (reprinted with permission from Ref. [25], MDPI, 2023). (**b**) Molecular structures of two anthraquinone-based azo dyes (15B and 26B) and schematic diagram of transition dipole directions of chromophores (**i**), dichroic ratio spectra of anthraquinone-based azo dye (**ii**), and cross-transmittances of the polarizing films (**iii**) (reprinted with permission from Ref. [36], Elsevier, 2012).

Because the chemical structure of a dye molecule affects the intrinsic dichroic performance, the design and optimization of dye molecular structures is one of the widely applied approaches to improve the performance of dye-based polarizers. Traditional dichroic dyes, especially the most widely used azo and anthraquinone dyes, although possessing a linear planar configuration, have inherent limitations such as a limited absorption wavelength range and primary reliance on hydrogen bonding with the PVA matrix film. This commonly leads to insufficient polarization efficiency, weakening of hydrogen bonds under high temperature and humidity, and poor durability for prepared complex films. In order to solve this problem, researchers have structurally modified dye molecules through chemical synthesis. One strategy is to construct rigid linear backbones, such as extending the azobenzene structure, or expand the molecular conjugation system. Such molecular modifications are beneficial for increasing the dichroic ratio and adjusting the absorption wavelength effectively, which would optimize spectral characteristics and color neutrality. Azo-type liquid crystal dyes with extended chromophore conjugation have been synthesized, resulting in high-order parameters and a complete color range [34]. The other method is to introduce functional groups, such as sulfonic acid groups and amino groups, into the molecule. They can not only enhance the water solubility of dye molecules to improve dyeing uniformity but also effectively adjust the electron cloud distribution of the dye molecule to optimize its optical anisotropy. These introduced polar or ionic groups can form stronger and more stable hydrogen bonds with the hydroxyl groups on the PVA chains. It is also helpful to enhance the heat and humidity stability of dye-based complex film due to the improved interfacial bonding force between the dye and PVA chains [35]. For example, Lee et al. [25] designed and synthesized a series of reactive dyes to solve this problem. In the traditional dye molecules, amino groups were replaced with cyanuric chloride groups to solve the problem of durability degradation due to hydrogen bond failure in direct dyes under high-humidity environments. After this modification, the dye can form strong covalent bonds with the PVA matrix (Figure 3(ai)). As shown in Figure 3(aii), the prepared films with reactive dyes significantly reduce the color difference value of the polarizer in the 85 °C/85% RH test and improve durability by over 50% without significantly sacrificing optical orientation ability. This demonstrates that constructing a covalent interface is an effective path for achieving stability in high-temperature and high-humidity environments.

In addition to the above strategies, constructing single-molecule structures with multiple chromophores, such as designing bis-azo or tris-azo structures, is another effective method. Such molecular designs can significantly broaden the absorption spectrum range while maintaining a linear configuration. Thereby, it can effectively improve the insufficient polarization ability of the polarizer at specific wavelengths, thereby enabling the device to exhibit a more ideal neutral color due to covering a wider visible light band [26]. For example, Chang et al. [36] designed and synthesized a series of dual-chromophore dyes combining anthraquinone and azo structures, systematically comparing their spectral and dichroic performance in PVA polarizing films with conventional linear bis-azo dyes. In synthesized dyes, the altered direction of the anthraquinone transition moment results in enhanced perpendicular absorbance, which directly accounts for the lower dichroic ratio observed in the shortwave region (Figure 3(bi)). Experimental results showed that the maximum dichroic ratios of synthesized anthraquinone-based azo dyes were obtained at longer wavelengths than their absorption maxima (Figure 3(bii)). Additionally, structural optimization of dye molecules also helps improve their optical properties. As shown in Figure 3(biii), the direct dyes typically absorbed in limited regions within the visible range. However, the absorption regions of the anthraquinone-based azo dyes were broader than that of direct dyes.

### 3.2. Optimization of Dyeing Process

Optimization of the dyeing process is also an indispensable key link in improving the performance of dye-based polarizers. One method is to optimize dye formulations to address the limitation of a single dye to overcome their limitations, such as a limited absorption band. This method involves mixing various dichroic dyes with complementary absorption bands to construct a broad-spectrum polarization absorption system covering ultraviolet to near-infrared ranges, which is able to synergistically improve the polarization efficiency and color neutrality of the polarizer. The selected dye components need to possess good dichroism, similar linear planar configurations, and polar characteristics to ensure highly consistent orientational alignment during the PVA stretching and film formation process, avoiding reduced order due to differences in molecular geometry or polarity. Furthermore, by systematically adjusting the ratio of each component, the polarization spectrum can be effectively broadened and the neutral gray tone precisely controlled while maintaining high dichroism.

**Figure 4:**
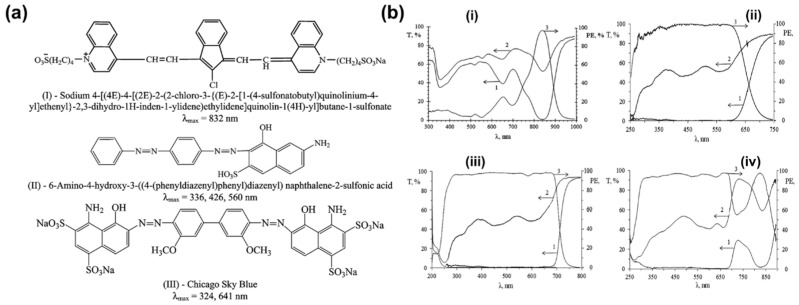
(**a**) Molecular structures of the three types of dichroic dyes; (**b**) transmission spectra (1-parallel, 2-perpendicular) and polarizing efficiencies (3-PE) of the broad band polarizing PVA films. Dichroic components (wt%): (**i**) 0.4 dye I; (**ii**) 0.2 dye II; (**iii**) 0.2 dye II + 0.2 dye III; (**iv**) 0.4 dye I + 0.2 dye II + 0.2 dye III (reprinted with permission from Ref. [37], Royal Society of Chemistry, 2016).

Several studies have reported dye formulations containing multiple dye types. Almodarresiyeh et al. [37] compounded a novel quinoline derivative (dye I, λmax = 832 nm) with two azo dyes absorbing in the visible region (dyes II and III). The chemical structures are shown in Figure 4a. This system utilized the quinoline dye to cover the near-infrared region and the azo dyes to synergistically cover the UV–Vis region, enabling the composite film to achieve a polarization efficiency of 97–99% in the UV–Vis region (300–695 nm) and 90–96% in the near-infrared region (819–855 nm). To create broad band polarizers operating in the spectral range of 300–900 nm, PVA films were colored by mixtures of the dyes (Figure 4b). Deeper compounding optimization has evolved from simple band superposition to fine control of the secondary absorption bands of dye molecules. Kato et al. [38] pointed out that the secondary absorption bands with relatively low dichroic ratios in dye molecules are key to limiting the performance of mixed systems. Based on this, they designed the new dyes B-IN and R-IN with high main absorption band absorbance and low secondary absorption band absorbance characteristics. After compounding them with traditional orange dyes, the dichroic ratio of the new polarizer reached 53, significantly better than the 35 of the traditional dye system. Furthermore, this optimized system exhibited outstanding durability in extreme environmental tests of 115 °C dry heat and 85 °C/85% RH conditions.

The dyeing process would also affect dichroic dyes ordered in a directional alignment and uniform distribution within the PVA matrix. By controlling process steps, such as stretching, dyeing, and temperature, it can synergistically enhance the polarization efficiency and transmittance of the polarizer [39]. By controlling the stretch ratio and temperature, the PVA polymer chains are fully extended and form an orientation template, which enables the adsorbed dye molecules to achieve consistent alignment along the stretching direction. Moreover, the dyeing process variables, such as dye concentration and immersion temperature, would effectively affect adsorption, penetration, and the uniform distribution of dye molecules in the PVA matrix. Compared to iodine-based polarizers, although dye-based polarizers have better resistance to heat and humidity, their optical performance strongly depends on the orientation state and interfacial bonding strength of the dye molecules within the PVA network. For example, researchers have reported that precisely controlling the dyeing temperature at 31–32 °C can significantly increase the dye uptake rate, reducing the dyeing time from 4 min to 1 min [40].

**Figure 5:**
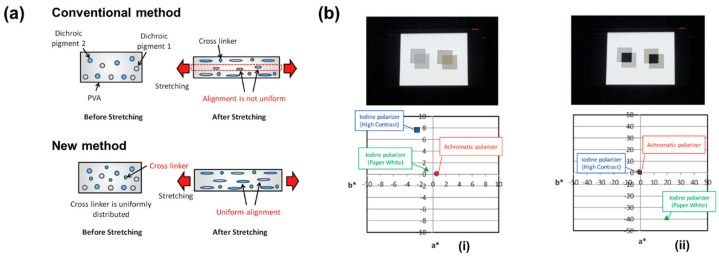
(**a**) Optimization of dyeing process to improve the orientational alignment of dichroic pigment through conventional and new methods; (**b**) photo and chromaticity of the polarizers in a (**i**) parallel state and (**ii**) crossed state. Left, achromatic polarizer; right, iodine-based polarizer (reprinted with permission from Ref. [8], ITE Transactions on Media Technology and Applications (MTA), 2018).

Furthermore, crosslinking and subsequent treatments are crucial for stabilizing the dye orientation structure and enhancing interfacial bonding. Varying concentrations of boric acid significantly affect the stretching structure of the films [41]. They would inhibit the crystallization of PVA during stretching and promote the formation of PVA–iodine complexes [42,43]. At low to medium concentrations of boric acid, the crystalline network is prone to fracture, with molecular chain slippage and nanofibril formation dominating the deformation process. Conversely, the crosslinking effect of boric acid maintains the connectivity of the crystalline network at high concentrations. This promotes early-stage orientation of molecular chains and the nucleation of nanofibrils but restricts their subsequent growth, ultimately resulting in a microstructure characterized by high orientation and low crystallinity. Furthermore, the stress–strain curves across different boric acid concentrations corroborate the regulatory role of boric acid in the microstructural transformation of PVA films. Additionally, since different dichroic dyes have different chemical structures, their migration and optimal alignment conditions in the PVA film can differ, which can lead to uneven alignment and overall achromatic issues. To solve the above problems, Mochizuki et al. [8] improved the crosslinking process by impregnating the dichroic dye and boric acid into the PVA film simultaneously before stretching, ensuring uniform distribution of boric acid through the film thickness (Figure 5a). This allowed for uniform stretching of the PVA film and highly consistent alignment of the pigment molecules in the subsequent boric acid aqueous solution stretching. As shown in Figure 5b, the transmittance of the achromatic polarizer is constant in the visible wavelength range, and the achromatic polarizer has small values of a* and b* compared to conventional high-contrast and paper-white iodine-based polarizers. The prepared dye-based polarizer with excellent achromatic performance, along with high brightness and dynamic display capability, is suitable for fields such as e-book readers and digital signage that require outdoor or long-duration display.

### 3.3. Modification of the PVA Film

In both dye-based and iodine-based polarizers, the PVA–based film serves as the matrix and orientation template for dye molecules, and its intrinsic characteristics significantly affect the final performance of the polarizer. For example, the molecular weight of PVA can affect the optical performance of dye-based polarizers. Lyoo et al. [27] prepared films using PVA with the same degree of alcoholysis and tacticity with different molecular weights and then prepared polarizers by dyeing with azo dyes. The results showed that as the PVA molecular weight increased, dye molecules more easily combined with the PVA chains to exhibit improved polarization performance, maintaining an optimal transmittance greater than 40%, while the DOP was greater than 99%. Furthermore, high-molecular-weight PVA molecules also made it difficult for dye molecules to be removed from the PVA film under high-temperature and high-humidity environments. When the polymerization degree was high (Mw~4000), the change in the DOP was only 1% under high-temperature and -humidity conditions for the dye-based polarizer. However, the high hydrolysis indicates a high content of hydrophilic hydroxyl groups on PVA molecular chains, which makes them prone to moisture absorption and swelling in humid environments. It would cause dimensional instability of the base film and reduce the mechanical properties in the wet state to disrupt the ordered arrangement of dye molecules and weaken their interfacial bonding force. It would restrict the polarizer’s ability to maintain high optical durability and a long service life in harsh environments. Some scenarios need to undergo operation under harsh conditions, such as in a surgery room with a daily disinfection process with high temperature and high humidity. In order to make these polarizer films to be stable under harsh conditions, it is necessary to modify the PVA–based film to improve its water resistance and mechanical stability. Additionally, the functionalization of PVA films would provide additional properties, including flame retardancy [44,45,46], water resistance [47,48,49], and UV shielding [50,51,52]. Current modification methods for PVA–based films can be mainly divided into chemical crosslinking modification, nanocomposite modification, and blending modification.

#### 3.3.1. Chemical Modification of PVA Chains

**Figure 6:**
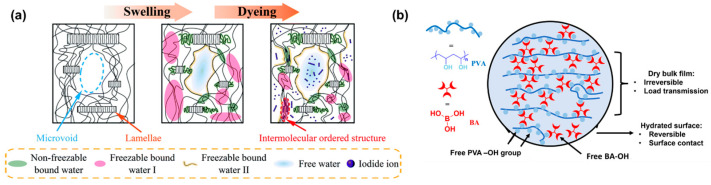
(**a**) The schematic mechanism of the dyeing processes for PVA films (reprinted with permission from Ref. [53], Royal Society of Chemistry, 2021); (**b**) illustration of PVA–boric acid crosslinking structure (reprinted with permission from Ref. [55], PNAS, 2022).

**Figure 7:**
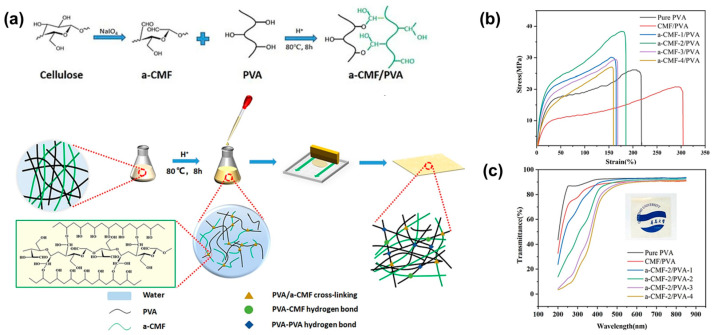
(**a**) Schematic diagram of the preparation of a-CMF/PVA composites; (**b**) stress–strain curves of a-CMF-2/PVA composite films containing various a-CMF-2 contents; (**c**) UV–vis transmittance spectra of PVA, CMF/PVA, and a-CMF-2/PVA composite films with various fiber contents (reprinted with permission from Ref. [60], Elsevier, 2022).

The core purpose of chemical modification is to build a stable covalent crosslinked network between PVA molecular chains to effectively restrict chain segment movement and reduce the number of free hydroxyl groups. In the dyeing process, iodide ions penetrate into the swollen PVA matrix, followed by the transformation from the loosely hydrated PVA segments to the intermolecular ordered structure (Figure 6a) [53]. After crosslinking, the film can exhibit a significantly enhanced resistance to moisture absorption, dimensional stability under heat and humidity, and mechanical strength, providing a more robust and stable orientation platform for dye molecules. Common cross-linkers include glutaraldehyde, boric acid and its derivatives, etc. Taking iodine-based polarizers as an example, boric acid is used as a classic cross-linker. This cross-linker can not only act as a coordinating agent for iodide ions in the PVA-I_2_ system but also react with the hydroxyl groups on PVA chains to form borate ester bonds. It would effectively enhance the stability of the PVA network to inhibit the dissociation of iodide ions under high humidity. Furthermore, researchers found that by optimizing the boric acid concentration and treatment process, moderate crosslinking could improve the durability of the polarizer by more than 30% under 85 °C/85% RH conditions without significantly sacrificing optical performance [54]. However, the crosslinking structure formed with boric acid is a dynamic structure (Figure 6b) [55]. Cross-linkers with bifunctional structures, such as glutaraldehyde and succinic acid [56,57,58], can be used to form a denser and more stable covalent crosslinked network, which is beneficial for improving optical performance, thermal stability, mechanical properties, and water resistance. For example, citric acid was used as a crosslinking agent to prepare PVA film to increase Young’s modulus and tensile strength due to the effects on crystallization [59]. The crosslinked network provided by citric acid also improved the thermal stability and reduced the water vapor permeability of composite film. Additionally, a type of PVA composite film was fabricated via a pre-crosslinking method with cellulose microfibril (CMF) [60]. The introduction of aldehyde groups (-CHO) onto the CMF surface significantly enhanced its reactivity with PVA, enabling the formation of a robust crosslinked network through aldol condensation between the aldehyde groups and PVA chains (Figure 7a). As shown in Figure 7b,c, this covalent crosslinking not only improved the interfacial compatibility and dispersion of the CMF within the PVA matrix but also synergistically enhanced the mechanical properties, UV-shielding capability, and hydrophobicity of the resulting films.

#### 3.3.2. Nanocomposite of PVA Film

**Figure 8:**
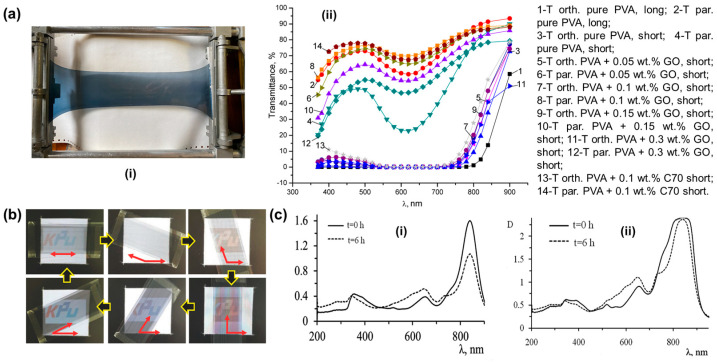
(**a**) A photo of the prepared polarizing film in a stretchable frame (**i**); the transmittance of the parallel (T par.) and orthogonal (T orth.) light components for the thin-film polarizers with a stretching of 3.5 times (long) and of 1.8 times (short) (**ii**) (reprinted with permission from Ref. [77], MDPI, 2024.). (**b**) Clearness of the letters on the screen according to the rotation angle of the CNT polarizer (reprinted with permission from Ref. [78], John Wiley and Sons, 2018.). (**c**) Optical density of the polarizing films incorporated with ZnO before and after UV radiation: (**i**) 0.4 wt% dye I and (**ii**) 0.4 wt% dye I + 1.0 wt% ZnO NPs (reprinted with permission from Ref. [37], Royal Society of Chemistry, 2016).

Nanocomposite modification primarily involves introducing inorganic nanofillers into the PVA matrix to enhance material properties by utilizing their interaction with PVA molecular chains. These inorganic nanofillers include nanodiamond [61,62,63], carbon dots [64,65,66], boron nitride [67,68,69], clay nanomaterials [70,71,72,73], and nanocellulose [60,74,75,76], which have rich oxygen-containing groups on their surfaces. These oxygen-containing groups easily form hydrogen bonds or covalent bonds with the hydroxyl groups on PVA chains to form stable structures. A polarizer film was prepared by incorporating graphene oxide (GO) into PVA films followed by direct stretching (as shown in Figure 8a) [77]. The results demonstrated that the incorporation of 0.1 wt.% GO enabled a parallel transmittance of up to 75%. The microhardness of the GO-doped PVA film was significantly higher than that of the pristine PVA film. With an increasing stretching ratio, the network constructed by GO presented a stronger orientational constraint on the PVA molecular chains, leading to a more pronounced enhancement in mechanical strength. However, an excessively high GO content (0.3 wt.%) deteriorated the surface structure of the PVA, causing a substantial reduction in parallel light transmittance, while an insufficient concentration failed to form an effective network, resulting in only a limited improvement in polarization performance. Additionally, research work also reported using carbon nanotubes (CNTs), which have anisotropic absorption within a wide spectral range, to prepare polymer composite film with uniform optical polarization performance in the visible light region (Figure 8b) [78]. The prepared PVA nanocomposite film with functionalized SWCNT exhibited improved mechanical properties due to good dispersion and strong interfacial bonding [79]. As a member of carbon-based nanomaterials, graphene quantum dots (GQDs) have also been widely used to prepare polymer–carbon composites to improve thermal stability and other comprehensive properties [80,81,82]. As zero-dimensional carbon nanomaterials, GQDs possess a high specific surface area, abundant oxygen-containing groups, excellent optical properties, and easy synthesis [82,83,84]. The UV absorption intensity of PVA/CQD composite films increases significantly with the increase in CQD concentration. This result indicates that the films are endowed with excellent UV-shielding performance compared to pure PVA, which also simultaneously enhances the mechanical properties and water resistance [65,66]. Additionally, the other types of incorporated nanomaterials could provide additional functions. Almodarresiyeh et al. [37] found that the PVA complex film could slow down the photodegradation process of dye molecules by adding 1 wt% ZnO nanoparticles to the PVA film. When original dyed PVA film was irradiated by UV light, the intensity at the absorption maximum decreased (Figure 8c). This observation suggests that the dichroic dye underwent photochemical transformation, resulting in the disruption of its conjugated double-bond system. While modifying the colored films with ZnO nanoparticles as an additional treatment, the films showed significantly enhanced light resistance.

**Figure 9:**
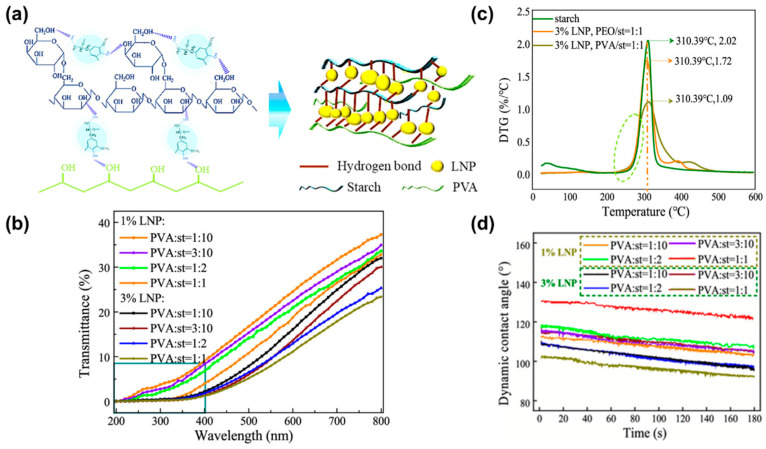
(**a**) Illustration of LNP distribution in the starch–PVA composite film and the hydrogen bonding; (**b**) transmittance, (**c**) DTG curves, and (**d**) dynamic contact angles of starch–PVA composite films with the addition of LNPs (reprinted with permission from Ref. [88], American Chemical Society, 2022).

Additionally, the incorporation of organic nanofillers into PVA films would also help improve comprehensive properties [85,86,87]. For example, a type of core–shell lignin nanoparticle (LNP) was added into the PVA film to achieve advanced performance [88]. As shown in Figure 9a, the introduction of LNPs created surface micro-roughness via the formation of micro-clusters, and starch–PVA hydrogen bonding improved interfacial compatibility and hydrophobic stability. At a starch/PVA ratio of (1:1) with LNPs, the film achieved robust UV shielding without sacrificing visible light transmittance (Figure 9b). Additionally, as shown in Figure 9c, 3 wt.% LNPs imparted high thermal stability (maximum degradation rate: 1.09%/°C) by suppressing H-O-H/O-H vibrations, reducing intermolecular hydrogen bonds, and forming a dense 3D network that inhibited thermal chain scission. These research works indicate that adding nanoparticles can disrupt the original hydrogen bonding in the PVA matrix, allowing PVA chains to move more freely. Simultaneously, nanoparticles can impart additional functionality to the PVA optical film. Moreover, the strong hydrogen bonds formed between nanoparticles and the PVA chains can act as bridge points to improve thermal stability and water resistance (Figure 9d).

#### 3.3.3. PVA–Polymer Blend Films

**Figure 10:**
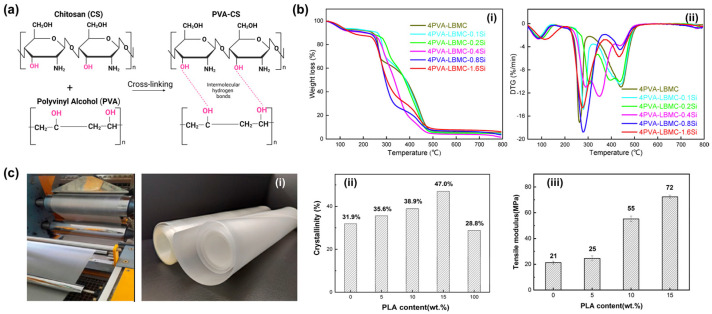
(**a**) Preparation process and thermal performance analysis of PVA–chitin film (reprinted with permission from Ref. [104], MDPI, 2023). (**b**) TGA (**i**) and DTG curves (**ii**) of the PVA/LBMC blend films (reprinted with permission from Ref. [89], Elsevier, 2020). (**c**) Melt blending of PVA/PLA and the obtained blend films (**i**); crystallinity (**ii**) and Young’s modulus of PVA/PLA blend system (**iii**) (reprinted with permission from Ref. [94], John Wiley and Sons, 2021).

Single-component polymers are often limited in practical applications due to their relatively simple chemical structure. Blending is one of the most direct and effective methods for modifying polymers. It can not only improve the physical properties of a single component but also endow the blend system with new structural and functional characteristics. For example, blending PVA with other polymers can prepare PVA blend films with high elasticity, mechanical strength, permeability, or water resistance. Depending on specific performance requirements, different polymers can be selected to compound with PVA, including chitosan (CS) [89,90,91,92,93], polylactic acid (PLA) [94,95,96,97], polyvinyl pyrrolidone [98,99,100], and starch [69,101,102,103]. These polymers contain a high content of oxygen-containing groups, and they can form hydrogen bonds with PVA to increase the crystallinity of the blend film or reduce the affinity of PVA hydroxyl groups for water. As a result, these polymer blends exhibited enhanced thermal stability and water resistance. For example, the combination of CS and PVA was shown to have good mechanical and chemical properties due to their unique intermolecular interactions based on their chemical structure and physical properties [104]. As shown in Figure 10a, PVA has a high degree of hydrogen bonding between its hydroxyl groups (-OH), while CS has amino groups (-NH_2_) in its chemical structure, which can form hydrogen bonds with the hydroxyl groups of PVA [104]. Zhang et al. [89] reported blending liquefied ball-milled chitin (LBMC) with PVA to obtain PVA/LBMC blend films. This hydrogen bond network formed between LBMC and PVA partially disrupts the ordered arrangement of PVA chains, significantly enhancing the intermolecular interaction forces, which is beneficial for reducing the affinity of free hydroxyl groups in PVA for water to improve the film’s water resistance. Additionally, it can significantly improve the thermal stability of polymer blends. As shown in Figure 10b, the maximum thermal decomposition temperature increased from 265 °C to 307 °C, and the weight loss rate reduced from 35% to 30%. The mechanical properties can be further enhanced by in situ introduction of SiO_2_ into the PVA/CS blend film [90]. Liu et al. [94] prepared PVA/PLA degradable blend films via melt blending (Figure 10(ci)). Structural characterization analysis confirmed the formation of hydrogen bonds between the ester groups (C=O) of PLA and the hydroxyl groups (–OH) of PVA. This interaction disrupted the original intramolecular and intermolecular hydrogen bond network of PVA, activated the PVA chain segment movement, and significantly improved the melt fluidity and processability of the blend system. As shown in Figure 10(cii), the formation of hydrogen bonds further induced the ordered arrangement of PVA molecular chains at the PLA phase interface, increasing the crystallinity of the blend film from 31.9% for pure PVA to 47% with 15 wt% PLA. The prepared PVA/PLA blends also showed an increased Young’s modulus, indicating enhanced mechanical properties (Figure 10(ciii)). Simultaneously, PLA, as a hydrophobic component, uniformly dispersed in the PVA matrix, would be useful to physically block water penetration to reduce the polymers’ affinity for water, significantly increasing the water contact angle from 23° to 60°, effectively improving the film’s water resistance.

Although the aforementioned research mostly focuses on non-polarizer application fields such as packaging and separation membranes, the blending mechanisms and structure–property relationships revealed therein have important reference value for the development of PVA–based films for polarizers. For example, the hydrogen-bond-regulated crystallization behavior and interface strengthening mechanism can be directly applied to optimize the optical properties. While maintaining good compatibility with the PVA substrate, it enhanced the hydrogen bonding or ionic bonding interaction with dye molecules, effectively improving the interaction between dye molecules and the PVA–based film to achieve the effect of increasing the PE. Meanwhile, the strategies for enhancing water resistance and thermal stability help alleviate the performance degradation of polarizers under high-temperature and -humidity environments. Therefore, systematically drawing on the achievements of the aforementioned chemical modification, nanocomposite modification, and blending modification of PVA films can provide new ideas and methodological support for promoting the functional design of high-performance dye-based PVA complex film as polarizers.

## 4. Summary and Discussion

Focusing on dye-based PVA complex film for optical applications, this article systematically reviews the relevant mechanistic content, compares diverse optimization approaches, and summarizes the effectiveness and mechanisms of different modification methods. To meet the diversified demands of emerging display technologies, including lightweight, flexible devices and stable operation under high humidity and temperature, dye-based PVA polarizers must evolve toward systems with higher contrast, improved environmental stability, and better process controllability. To advance this field, several research directions can be proposed. (1) First, the structure–property relationship of dichroic dyes should be systematically investigated. Key controlled variables include dye molecular conjugation length, substituent polarity, and functional groups capable of hydrogen bonding with PVA chains. Success metrics may include increased dichroic ratio, higher polarization efficiency, and improved photostability. It is expected that optimized dye structures with stronger intermolecular interactions and anisotropic absorption will improve dye alignment and optical performance. (2) Second, the formulation and processing strategies used during polarizer fabrication should be optimized to enhance dye adsorption and alignment consistency. Controlled variables may include the dye concentration, co-dye formulation, stretching ratio, and processing temperature. It is expected that optimized processing windows will improve dye orientation and reduce structural defects to achieve improved polarization efficiency. (3) Third, improving the PVA–based film represents an underexplored but promising strategy for enhancing the comprehensive performance of dye-based polarizers. Controlled variables include the PVA molecular weight, degree of hydrolysis, tacticity, and crosslinking density. These strategies may benefit to improve water resistance, thermal stability, and long-term optical stability while maintaining high polarization efficiency. It is expected that optimized PVA microstructure and interfacial interactions will enhance dye fixation and overall durability. (4) In addition, data-driven molecular design approaches, such as hierarchical molecular representation frameworks (HiFrAMes) [104], may provide new opportunities for accelerating the discovery of high-performance dichroic dyes and establishing quantitative structure–processing–performance relationships in dye-based polarizers. These directions highlight the importance of systematically linking molecular design, processing conditions, and macroscopic optical performance, which will support the development of next-generation high-performance dye-based PVA complex films for advanced optical and display applications.

## Figures and Tables

**Table 1:** Summary of the properties of iodine-based and dye-based PVA complex films [28].

	Optical Properties		Stability	
	Transmittance	PE	Mimic Sunlight ^a^	85 °C/85% RH ^b^
Iodine-based	41.5%	99.99%	<500 h	<240 h
Dye-based	39.0%	99.70%	>1000 h	>1000 h

^a^ The time to keep the hue value change within 1% reduction based on original values under a mimicked sunlight condition (Xenon light). ^b^ The time to keep the transmittance and PE within 5% reduction under an 85 °C/85% RH condition.

## Data Availability

No new data were created or analyzed in this study.
